# Ca^2+^-Independent Short-Term Depression in the Hippocampal CA1 Region

**DOI:** 10.3390/ijms27156908

**Published:** 2026-08-01

**Authors:** Margarita A. Novikova, David Jappy, Dmitrii A. Fedorov, Elizaveta A. Klimanova, Georgii M. Nikolaev, Irina A. Korneeva, Olga D. Lopina, Olga A. Averina, Andrei Rozov, Alexander V. Latanov

**Affiliations:** 1 Faculty of Biology, Lomonosov Moscow State University, Moscow 119234, Russia; novikova.margarita2011@gmail.com (M.A.N.); fedorovdmitry42@gmail.com (D.A.F.); gnikolaev@neurobiology.ru (G.M.N.); irina.korneeva1212@gmail.com (I.A.K.); od_lopina@mail.ru (O.D.L.); latanov@neurobiology.ru (A.V.L.); 2 A.N. Belozersky Institute of Physico-Chemical Biology, Lomonosov Moscow State University, Moscow 119992, Russia; averina.olga.msu@gmail.com; 3 Federal Center of Brain Research and Neurotechnologies, Federal Medical and Biological Agency, Moscow 117513, Russia; djjappy@hotmail.co.uk (D.J.); rozov1511@gmail.com (A.R.); 4 Research Institute for Brain Development and Peak Performance, Peoples’ Friendship University of Russia, Moscow 117198, Russia

**Keywords:** Ca^2+^-independent plasticity, CA1, short-term depression, hippocampus, non-synaptic plasticity, theta-burst stimulation

## Abstract

Ca^2+^ influx is usually considered a central trigger of neuronal plasticity, especially in mechanisms related to transmitter release and synaptic modification. However, plasticity may also involve mechanisms that do not require Ca^2+^ influx into neurons. We tested whether short-term plasticity can occur in the CA1 region of the mouse hippocampus under Ca^2+^-free conditions. In Ca^2+^-free artificial cerebrospinal fluid, in which synaptic transmission was not detected, local stimulation evoked a population potential in CA1, and theta-burst stimulation led to short-term depression during the stimulation protocol. This depression included three separable components: depression within bursts, cumulative depression of the first response across bursts, and enhancement of within-burst depression during repeated bursts. Whole-cell current-clamp recordings showed that the TBS stimulation protocol also changed action-potential kinetics at the level of individual neurons. These changes were expressed in CA1 pyramidal neurons as a reduction of maximum rise rate and slowing of repolarization, whereas *stratum oriens* interneurons did not show within-burst depression nor enhancement. Together, these data show that theta-burst stimulation can induce Ca^2+^-influx-independent short-term plasticity of action potentials in hippocampal CA1 pyramidal cells.

## 1. Introduction

Synaptic plasticity is widely considered one of the cellular mechanisms that supports learning and memory [1]. At the synaptic level, plasticity can involve presynaptic changes in neurotransmitter release and postsynaptic changes in receptor function or number. However, various forms of learning and patterns of neuronal activity can also be mediated by non-synaptic changes [2].

Neurons of the central nervous system possess powerful information-processing capabilities that are fundamental to sensory perception and motor control. They can accurately encode and transmit diverse spatiotemporal signals, initiating specific sequences of action potentials. However, neuronal coding can be modified by activity-dependent changes in conduction velocity and by disruptions in action-potential generation [3]. Importantly, local changes within a single neuron, rather than alterations that occur at the level of the neuronal synaptic network, can underlie these modifications in encoding and the formation of a specific activation pattern.

Non-synaptic plasticity of neurons may play a role in signal extraction, response selectivity, high-frequency filtering, signal-to-noise separation, and precise temporal coding, endowing the neuron with flexible functionality under diverse conditions [4].

Since non-synaptic plasticity primarily manifests as changes in action-potential waveform and/or action-potential firing patterns, the key mechanisms underlying this type of plasticity involve the regulation of ion channels participating in action-potential generation and propagation. Such changes are important because they determine how neuronal inputs are transformed into action-potential output. Although ion channel modulation is often considered in relation to Ca^2+^-dependent signaling, repeated activity may also change neuronal output through mechanisms that do not require Ca^2+^ influx into the neuron. In this study, we tested whether theta-burst stimulation can evoke short-term changes in CA1 population potentials and in action-potential kinetics in individual neurons under Ca^2+^-free conditions.

## 2. Results

### 2.1. Population Potential in Ca^2+^-Free ACSF Depends on the Activation of Voltage-Gated Na^+^ Channels

**Figure 1:**
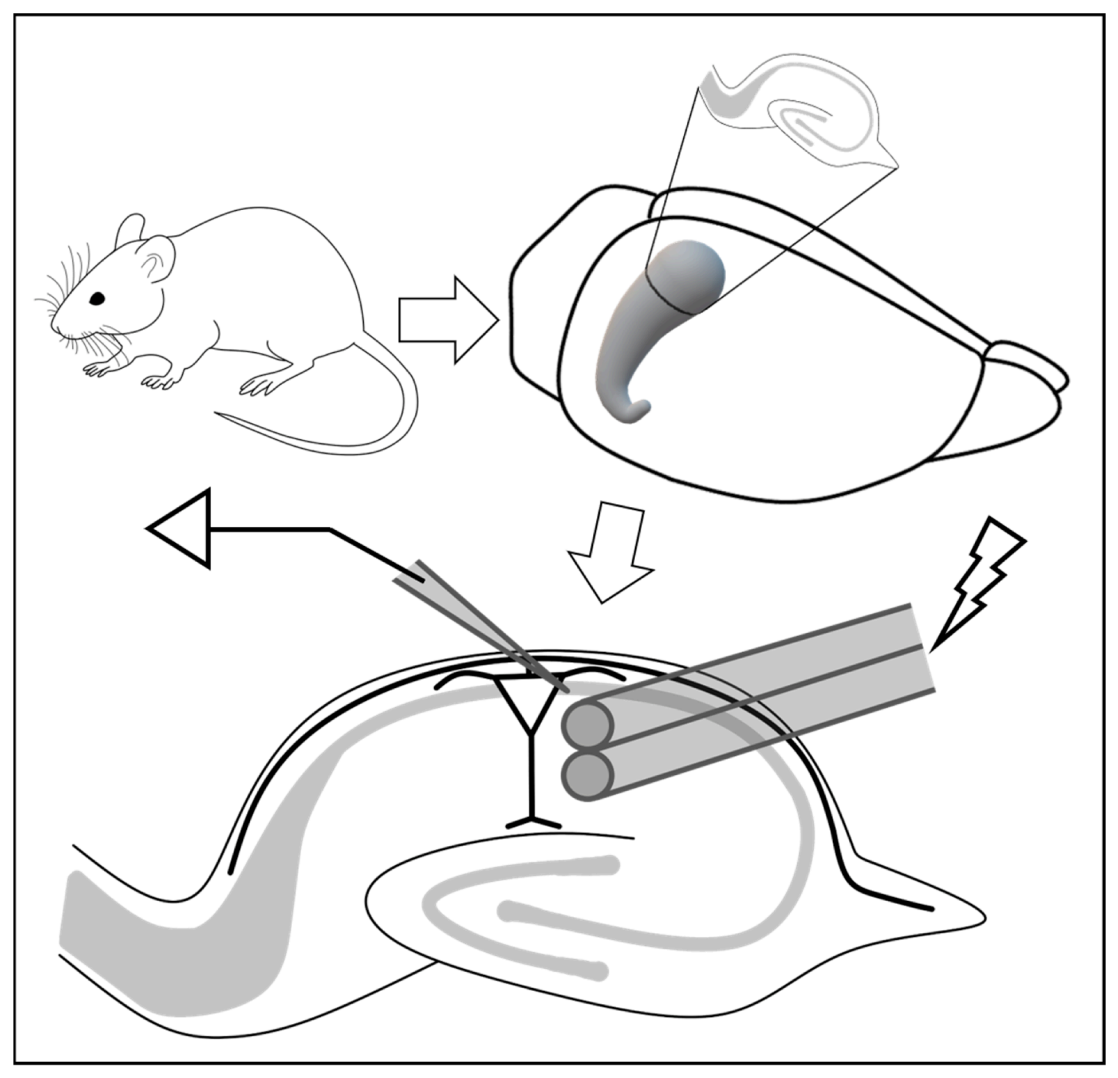
Schematic representation of slice preparation and electrode placement in the hippocampal slice. A bipolar stimulating electrode was placed in *stratum radiatum* of CA1, while a recording electrode was placed in *stratum pyramidale* of CA1 in close proximity to the stimulating electrode.

The aim of our study was to investigate neuronal plasticity independent of Ca^2+^ influx into the neuron. Therefore, after transferring the slices to the experimental chamber for further experiments, we used a Ca^2+^-free artificial cerebrospinal fluid solution containing the Ca^2+^ chelator EGTA (ethylene glycol tetraacetic acid). To ensure that all free Ca^2+^ ions were washed out of the slice and would no longer be able to affect neuronal plasticity, we stimulated the Schaffer collateral axons in the *stratum radiatum* at the border of the CA2 and CA3 fields and recorded population potentials in the CA1 field (Figure 1). Using a Ca^2+^-free solution, we did not observe population excitatory postsynaptic potentials in *stratum radiatum* nor population potentials in *stratum pyramidale* of the CA1 field. Thus, at the time of recording neuronal population activity, the concentration of Ca^2+^ ions in the extracellular solution was insufficient for the exocytosis of synaptic vesicles and the release of neurotransmitter into the synaptic cleft.

**Figure 2:**
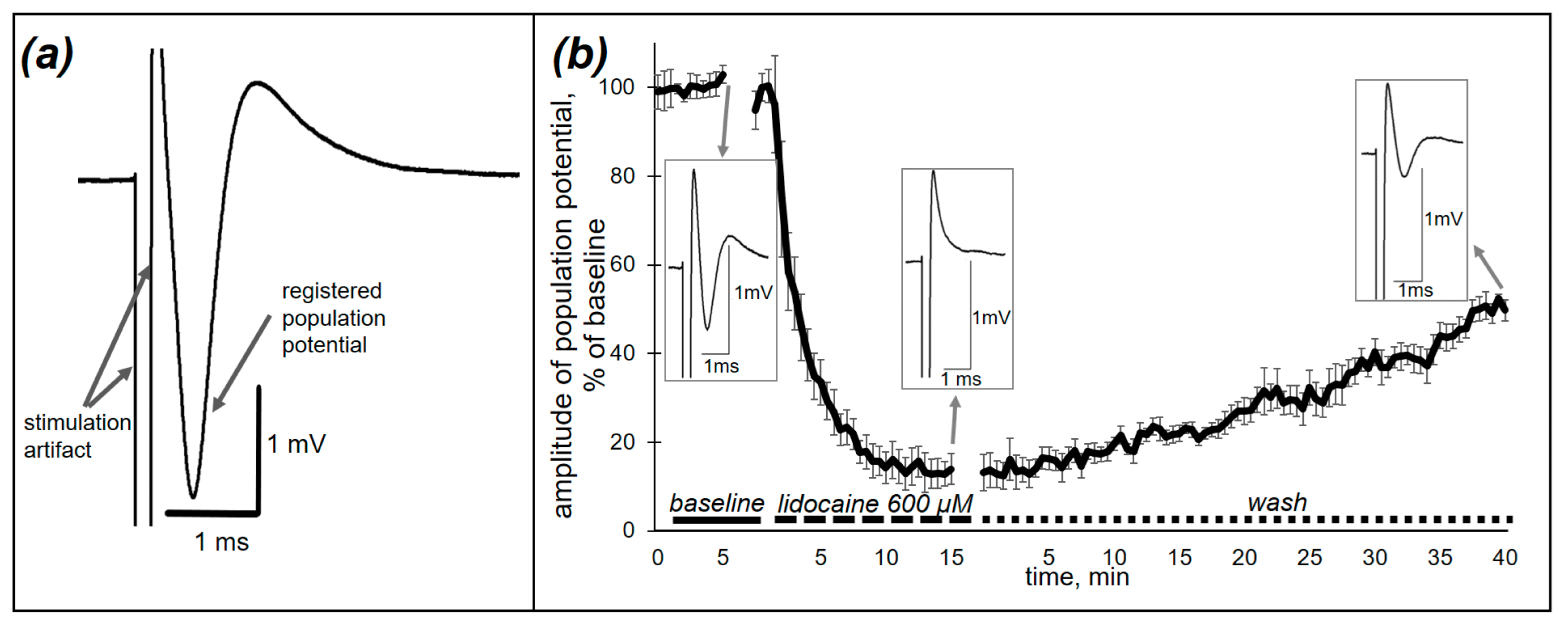
Population potential recorded in Ca^2+^-free ACSF (artificial cerebrospinal fluid) depends on the activation of voltage-gated Na^+^-channels. (**a**) Representative evoked population potential recorded in Ca^2+^-free ACSF. The trace is averaged over 20 sweeps. (**b**) Effect of 600 μM lidocaine on the amplitude of the population potential recorded in Ca^2+^-free ACSF. Data are presented as mean ± SEM, n = 5. Representative traces are shown in the insets and are averaged over 10 sweeps.

Since synaptic transmission was impaired under the conditions of our experiment, we hypothesized that the population response of neurons could only be detected upon direct stimulation with currents propagating electrotonically and depolarizing the membrane. As the hippocampal slice has some capacitive and resistive characteristics, to detect the population response of neurons to stimulation, the recording and stimulating electrodes must be located a small distance from each other. Using electrical stimulation of CA1 *stratum radiatum* in mouse acute hippocampal slices, we detected an evoked population potential in CA1 *stratum pyramidale* in the absence of extracellular Ca^2+^ ions (Figure 2a). The resulting signal was in close proximity to the stimulation artifact and was characterized by a fast decay time of 1–2 ms.

Since the recorded population potential could not occur due to the response to neurotransmitter release and the activation of ligand-gated ion channels (e.g., AMPA and NMDA receptors), we hypothesized that it was induced directly by a change in the membrane potential due to the propagation of current from stimulation. A change in the membrane potential of a neuron can lead to the activation of voltage-gated ion channels, in particular, voltage-gated Na^+^ channels. Therefore, we assumed that the recorded population potential depends on their activation. To test this hypothesis, we blocked voltage-gated Na^+^ channels with lidocaine. Here and further, for baseline stimulation, presumably not inducing neuronal plasticity, we chose a stimulation intensity equal to the strength at which the amplitude of the population potential was 40–60% of the maximum (determined visually based on stimulation with a strength of 100 mV), and performed stimulation with pulses with a duration of 0.1 ms every 30 s. Upon application of 600 μM lidocaine, the amplitude of this potential decreased to 14 ± 3.7% (mean ± standard error of the mean) of the baseline value. After 40 min of lidocaine washout, the amplitude of the response recovered to 47.7 ± 2.5% (mean ± standard error of the mean) of the baseline value (Figure 2b). The results obtained indicate that the recorded population potential depends on the activation of voltage-gated Na^+^ channels.

### 2.2. After Theta-Burst Stimulation, Post-Stimulation Changes in the Amplitude of the Population Potential Are Not Observed

We asked whether theta-burst stimulation could evoke post-stimulation or short-term changes in the Ca^2+^-free population potential under conditions in which synaptic transmission was not detected. To test our assumption, we used the following theta-burst stimulation (TBS) protocol: 10 bursts of pulses, with 4 pulses per burst at 100 Hz and an interburst interval of 200 ms. This stimulation pattern imitates two characteristics of hippocampal physiology: complex spikes of pyramidal neurons and rhythmic modulation of the excitability of these cells during the theta-rhythm. This protocol is used to induce long-term potentiation and elicits long-term plasticity that is more sensitive to experimental manipulations than that elicited by other induction protocols (e.g., high-frequency stimulation at 100 Hz for 1 s) [5].

**Figure 3:**
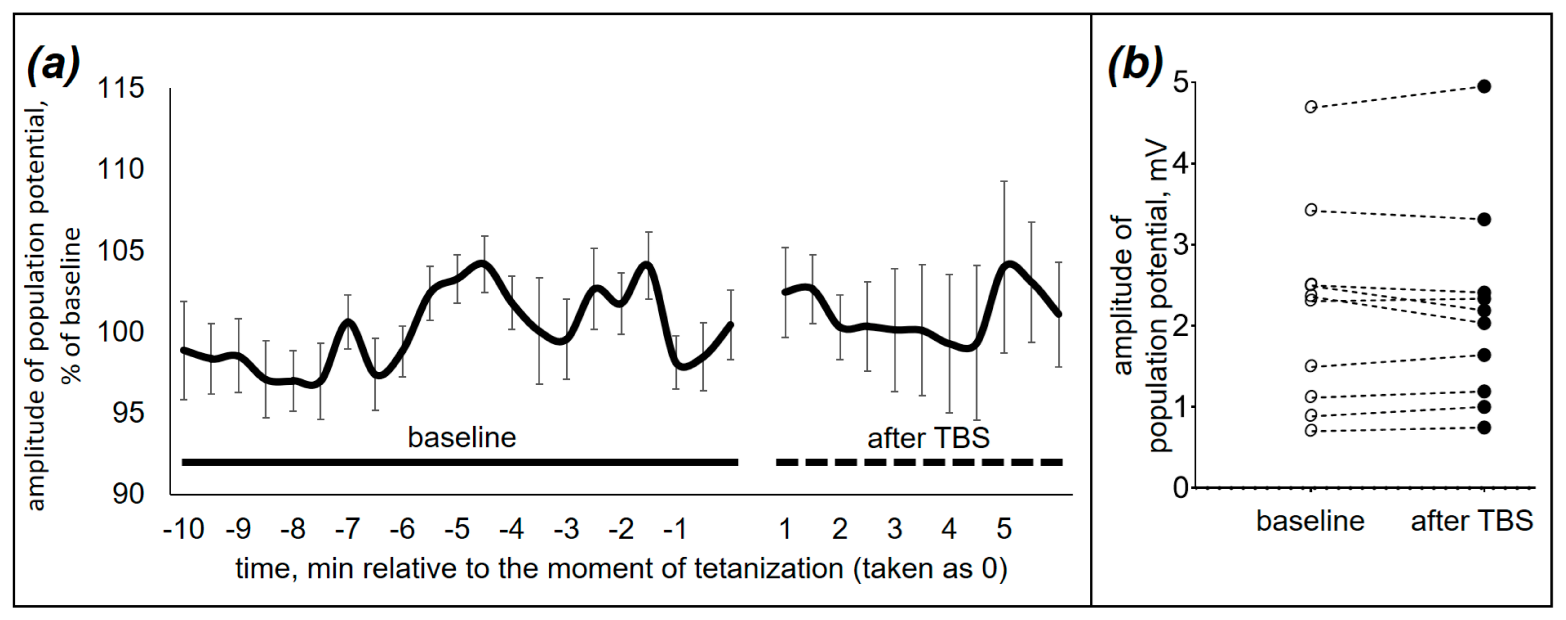
The TBS protocol did not induce changes in the amplitude of the population potential recorded in Ca^2+^-free ACSF. (**a**) Time course of the population potential amplitude during baseline recording and after TBS. The moment of TBS was taken as 0. Data are normalized to the mean baseline value and presented as mean ± SEM, n = 10. (**b**) Individual paired values of the population potential amplitude during baseline recording and after TBS. Baseline values were averaged over 20 sweeps, and post-TBS values were averaged over 10 sweeps. Statistical analysis was performed using a paired *t*-test.

We hypothesized that after using the TBS protocol, we would observe a long-term change in the amplitude of the population potential, similar to that observed with the classical method of recording population potentials in a Ca^2+^-containing solution. However, after using the TBS protocol, we did not observe any changes in the amplitude of the response over 5 min (Figure 3a,b).

### 2.3. During Theta-Burst Stimulation, Several Forms of Short-Term Neuronal Plasticity Occur Independently of Ca^2+^ Influx into Neurons

**Figure 4:**
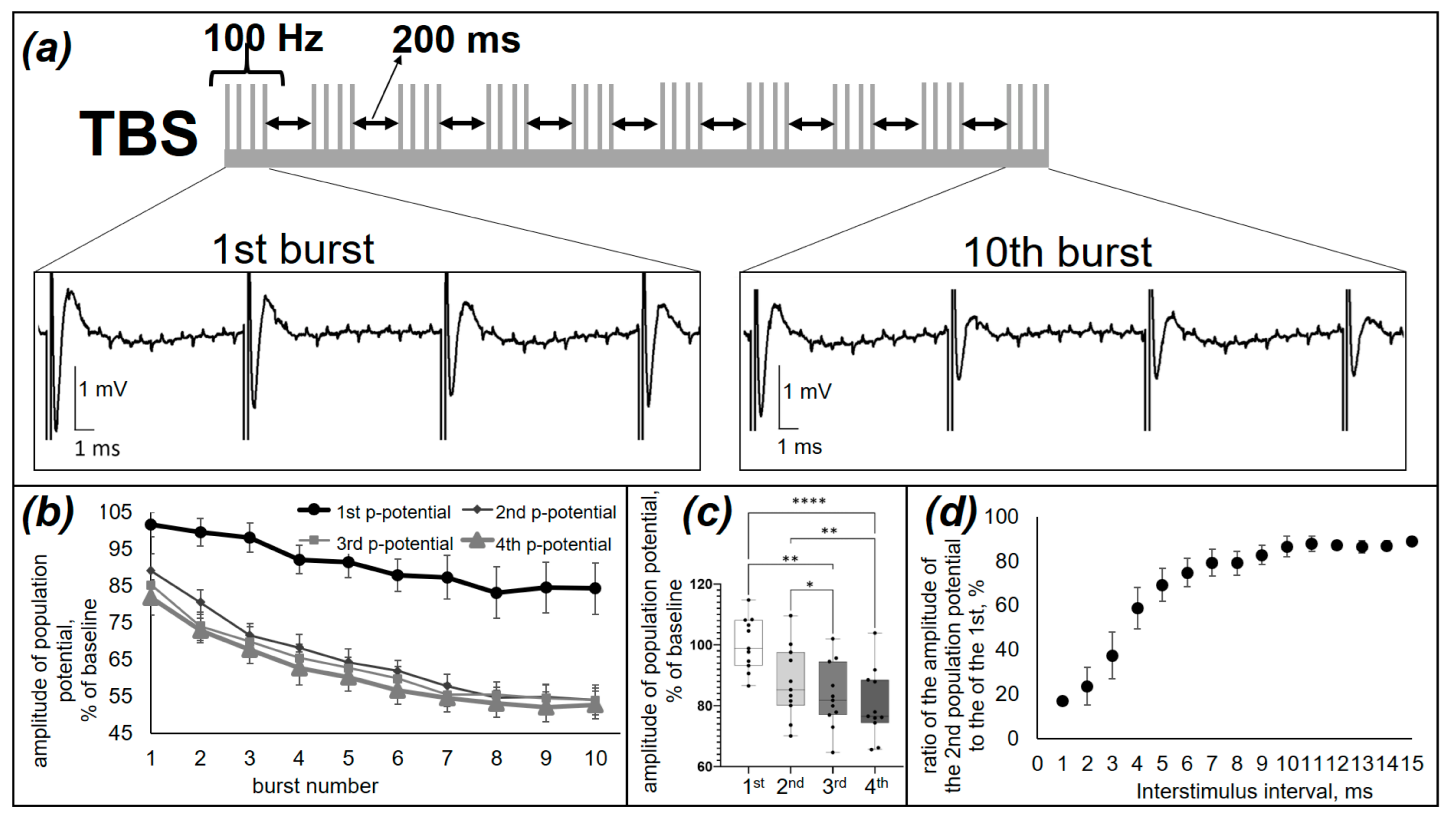
Short-term depression of the population potential during the TBS protocol in Ca^2+^-free ACSF. (**a**) Schematic representation of the TBS protocol and representative responses recorded during the 1st and 10th bursts. (**b**) Time course of the amplitude of the 1st–4th population potentials within bursts during TBS. Data are normalized to the baseline value and presented as mean ± SEM, n = 11. (**c**) Summary of the amplitudes of the 1st–4th population potentials within the 1st burst. Box plots show the median and interquartile range; whiskers indicate the range, and individual points represent individual slices. Statistical analysis was performed using the Friedman test followed by the two-stage linear step-up procedure of Benjamini, Krieger and Yekutieli; * q < 0.05, ** q < 0.01, **** q < 0.0001 for the indicated comparisons. (**d**) Dependence of the ratio of the amplitude of the 2nd population potential to the 1st population potential on the interstimulus interval. Data are presented as mean ± SEM, n = 5.

It was noted earlier that short-term plasticity may occur during high-frequency stimulation itself and that changes in this short-term plasticity may predict subsequent long-term potentiation [6]. Therefore, we hypothesized that short-term changes might be observed within the responses to a burst of pulses under our conditions. We found a decrease in the amplitude of the population potential both within a burst and in the 1st potential in the burst throughout the duration of high-frequency stimulation (Figure 4a–c).

We hypothesized that the decrease in the amplitude of the population potential within a burst may be related to the refractoriness of voltage-gated Na^+^ channels. To test this assumption, we used a paired-pulse protocol and varied the interstimulus interval from 15 to 1 ms. The amplitude of the second population potential was lower than the amplitude of the 1st potential at all interstimulus intervals. However, the pronounced interval-dependent decrease was observed only at intervals less than or equal to 9 ms (Figure 4d). Thus, the short-term depression of the population potential within a burst of pulses is unlikely to be explained only by refractoriness.

**Figure 5:**
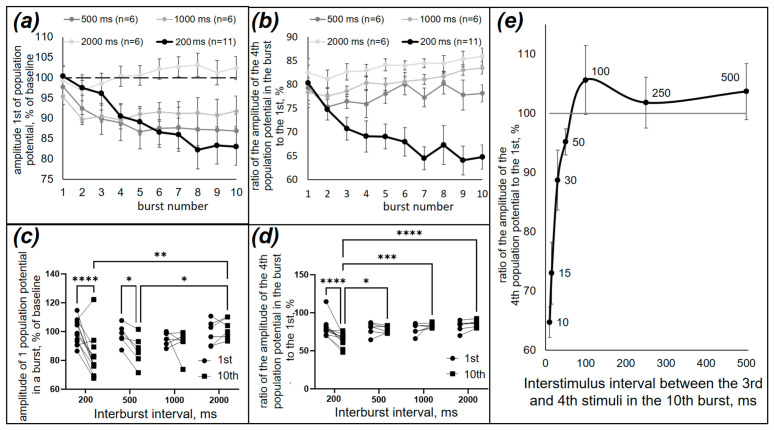
Effect of the interburst interval on Ca^2+^-independent short-term changes in population potentials during the TBS protocol. (**a**,**b**) Time course of the amplitude of the 1st population potential in each burst (**a**) and the ratio of the amplitude of the 4th population potential to the 1st population potential within each burst (**b**) during TBS protocols with different interburst intervals. Data are presented as mean ± SEM. (**c**,**d**) Individual values of the amplitude of the 1st population potential (**c**) and the ratio of the amplitude of the 4th population potential to the 1st population potential (**d**) in the 1st and 10th bursts for each interburst interval. (**e**) Dependence of the ratio of the amplitude of the 4th population potential to the 1st population potential in the 10th burst on the interstimulus interval between the 3rd and 4th stimuli. Statistical analysis for panels (**c**,**d**) was performed using two-way repeated-measures ANOVA followed by Tukey’s multiple comparisons test. * *p* < 0.05, ** *p* < 0.01, *** *p* < 0.001, **** *p* < 0.0001.

To understand the temporal characteristics of the short-term depression of the 1st potential in a burst, we varied the interburst interval in the range of 200–2000 ms. Under these conditions, we recorded a statistically significant decrease in the amplitude of the 1st potential in the 10th burst compared to the 1st burst only at interburst intervals of 200 and 500 ms (Figure 5a,c). This depression was cumulative, and the amplitude of the 1st potential in the burst reached a plateau by the 6th burst of stimulation (Figure 5a).

We observed that the kinetics of changes in the amplitude of the 1st potential within a burst differed from the kinetics of changes in the amplitude of subsequent potentials. The decrease in the amplitudes of the 2nd–4th potentials from the 1st to the 10th burst was more pronounced than the decrease in the amplitude of the 1st potential (Figure 4b). Therefore, we compared the ratio of the amplitude of the 4th potential to the 1st potential within a burst. The depression of the potential within a burst also changed during the TBS protocol (Figure 5b,d,e), indicating a metaplastic enhancement of within-burst depression. To study the temporal characteristics of this metaplastic change, we varied the interburst interval in the range of 200–2000 ms, as well as the interstimulus interval between the 3rd and 4th stimuli in the 10th burst. A statistically significant metaplastic change was observed only at an interburst interval of 200 ms (Figure 5b,d). Furthermore, when the interstimulus interval between the 3rd and 4th stimuli was increased from 10 to 100 ms, the depression gradually weakened and then disappeared. With a further increase in the interstimulus interval, the amplitude of the 4th potential did not decrease relative to the 1st potential (Figure 5e).

Thus, during the TBS protocol, we observed three Ca^2+^-independent short-term plasticity phenomena in population potentials: depression of the population potential within a burst, depression of the 1st population potential in a burst, and metaplastic enhancement of within-burst depression.

### 2.4. During Theta-Burst Stimulation in Ca^2+^-Free ACSF, Action-Potential Rise Rate Decreases and Repolarization Slows

To determine whether the short-term plasticity observed in population potentials is accompanied by depression of action-potential kinetics in individual cells, we performed whole-cell current-clamp recordings in Ca^2+^-free ACSF.

The action-potential waveform reflects the coordinated activity of voltage-gated ion channels, and its rising and repolarizing phases characterize the kinetics of depolarization and repolarization [7,8]. Therefore, to assess short-term depression of action-potential kinetics during the TBS protocol, we focused on the maximum rise and decay rates of action potentials.

**Figure 6:**
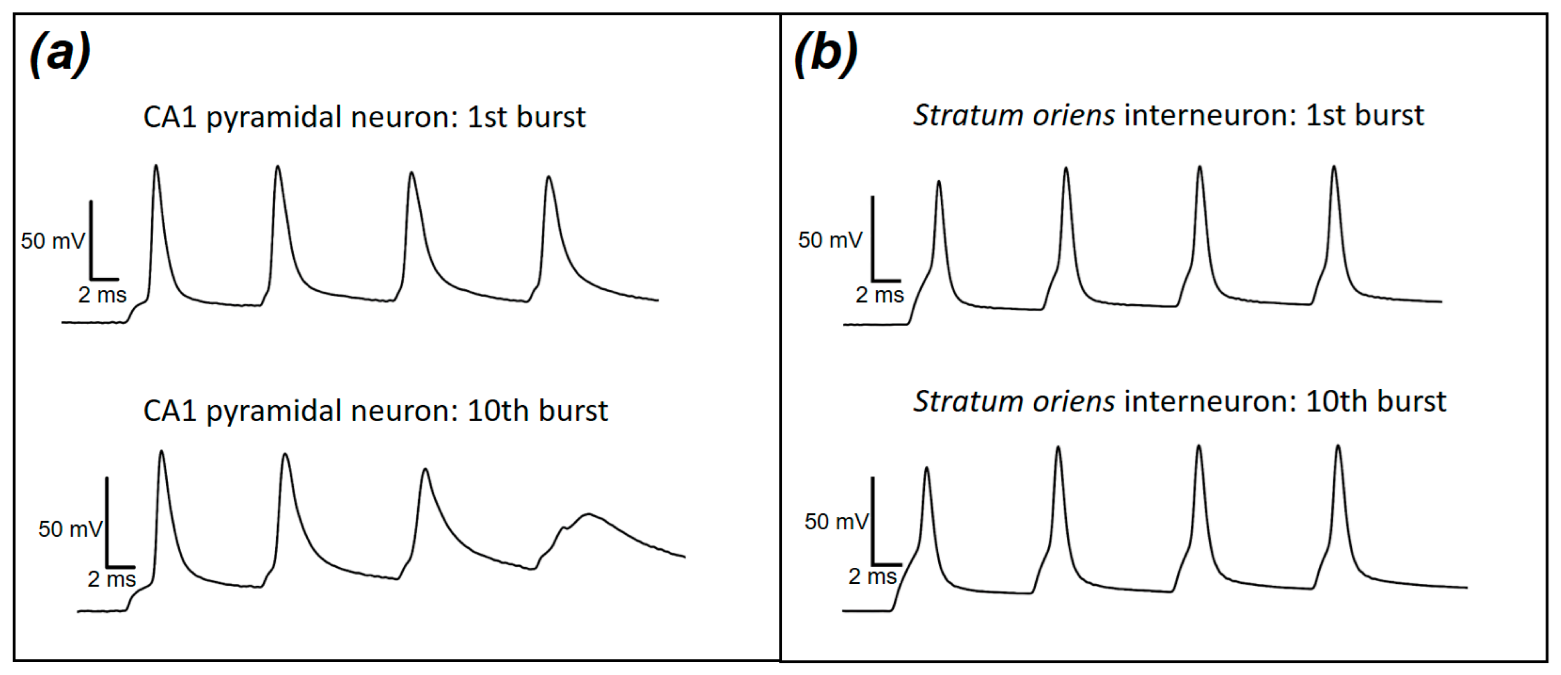
Representative whole-cell current-clamp recordings from CA1 pyramidal neurons and stratum oriens interneurons during the TBS protocol. (**a**) Representative action-potential recordings from a CA1 pyramidal neuron showing short-term changes during the TBS protocol. (**b**) Representative action-potential recordings from a stratum oriens interneuron without evident within-burst depression or enhancement of within-burst depression from the 1st to the 10th burst. Representative traces are shown for responses evoked within bursts. Scale bars are shown on the traces.

First, we recorded responses from CA1 pyramidal neurons during the TBS protocol (Figure 6a). We then recorded *stratum oriens* interneurons using the same stimulation protocol (Figure 6b). Further analysis compared action-potential kinetics in CA1 pyramidal neurons and *stratum oriens* interneurons.

**Figure 7:**
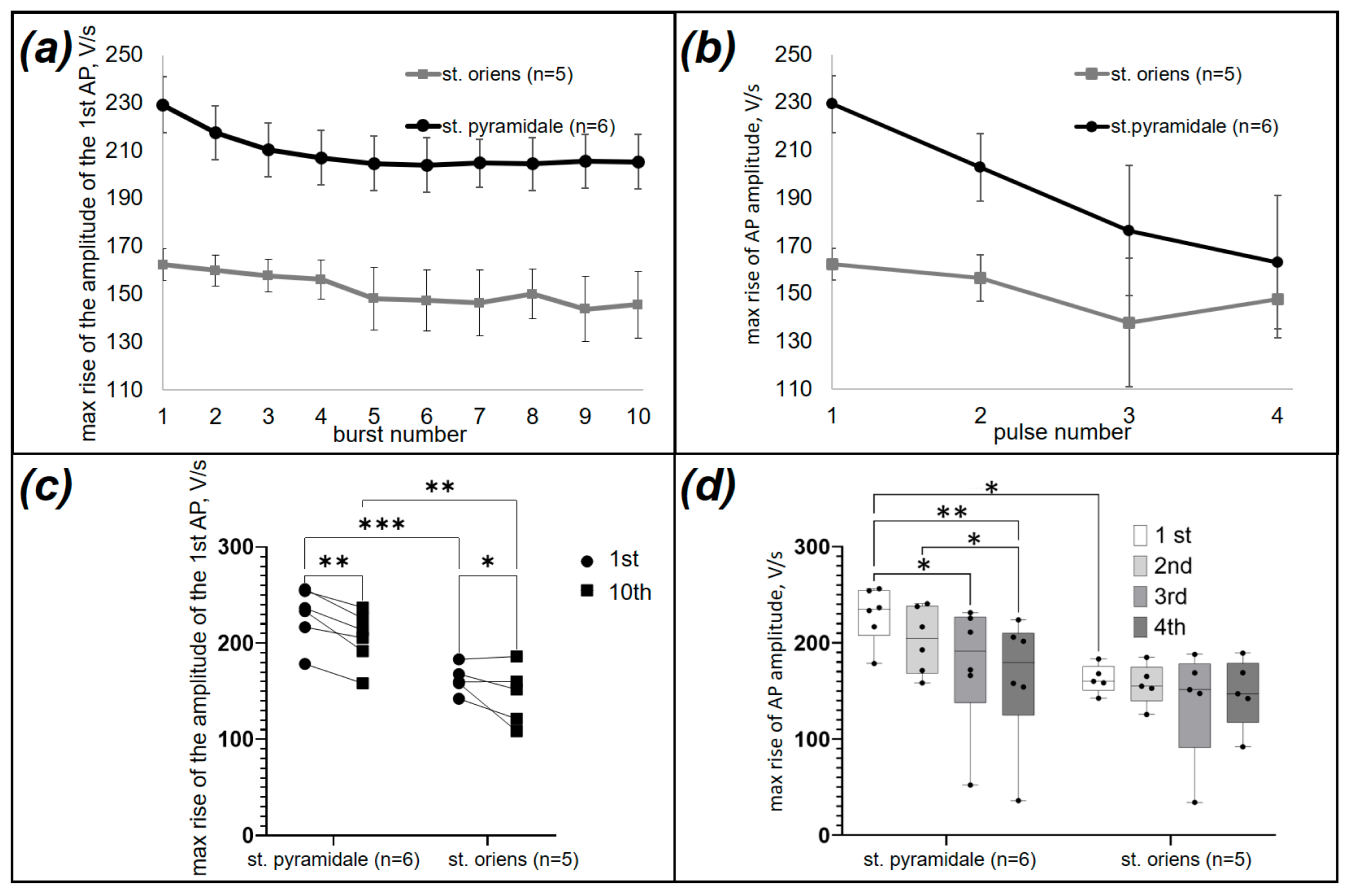
Changes in the maximum rise rate of action potentials in CA1 pyramidal neurons and *stratum oriens* interneurons during the TBS protocol. (**a**) Time course of the maximum rise rate of the 1st action potential across bursts in cells recorded in stratum pyramidale and stratum oriens. (**b**) Changes in the maximum rise rate of action potentials within a burst. Data in (**a**,**b**) are presented as mean ± SEM. (**c**) Individual paired values of the maximum rise rate of the 1st action potential in the 1st and 10th bursts in cells recorded in stratum pyramidale and stratum oriens. Statistical analysis for panel (**c**) was performed using two-way repeated-measures ANOVA followed by uncorrected Fisher’s LSD test; * *p* < 0.05, ** *p* < 0.01, *** *p* < 0.001 for the indicated comparisons. (**d**) Summary of the maximum rise rate of the 1st–4th action potentials within a burst in cells recorded in stratum pyramidale and stratum oriens. Box plots show the median and interquartile range; whiskers indicate the range, and individual points represent individual cells. Statistical analysis for panel (**d**) was performed using two-way repeated-measures ANOVA followed by the two-stage linear step-up procedure of Benjamini, Krieger and Yekutieli; * q < 0.05, ** q < 0.01 for the indicated comparisons.

In CA1 pyramidal neurons, the maximum rise rate of the 1st action potential decreased from the 1st to the 10th burst (Figure 7a,c). In *stratum oriens* interneurons, a decrease in the first-response maximum rise rate was observed in 3 of 5 cells (Figure 7c). At both time points, the maximum rise rate was higher in CA1 pyramidal neurons than in *stratum oriens* interneurons (Figure 7c). Within a burst, depression of the maximum rise rate was detected in CA1 pyramidal neurons, whereas no significant within-burst depression was detected in *stratum oriens* interneurons (Figure 7b,d).

**Figure 8:**
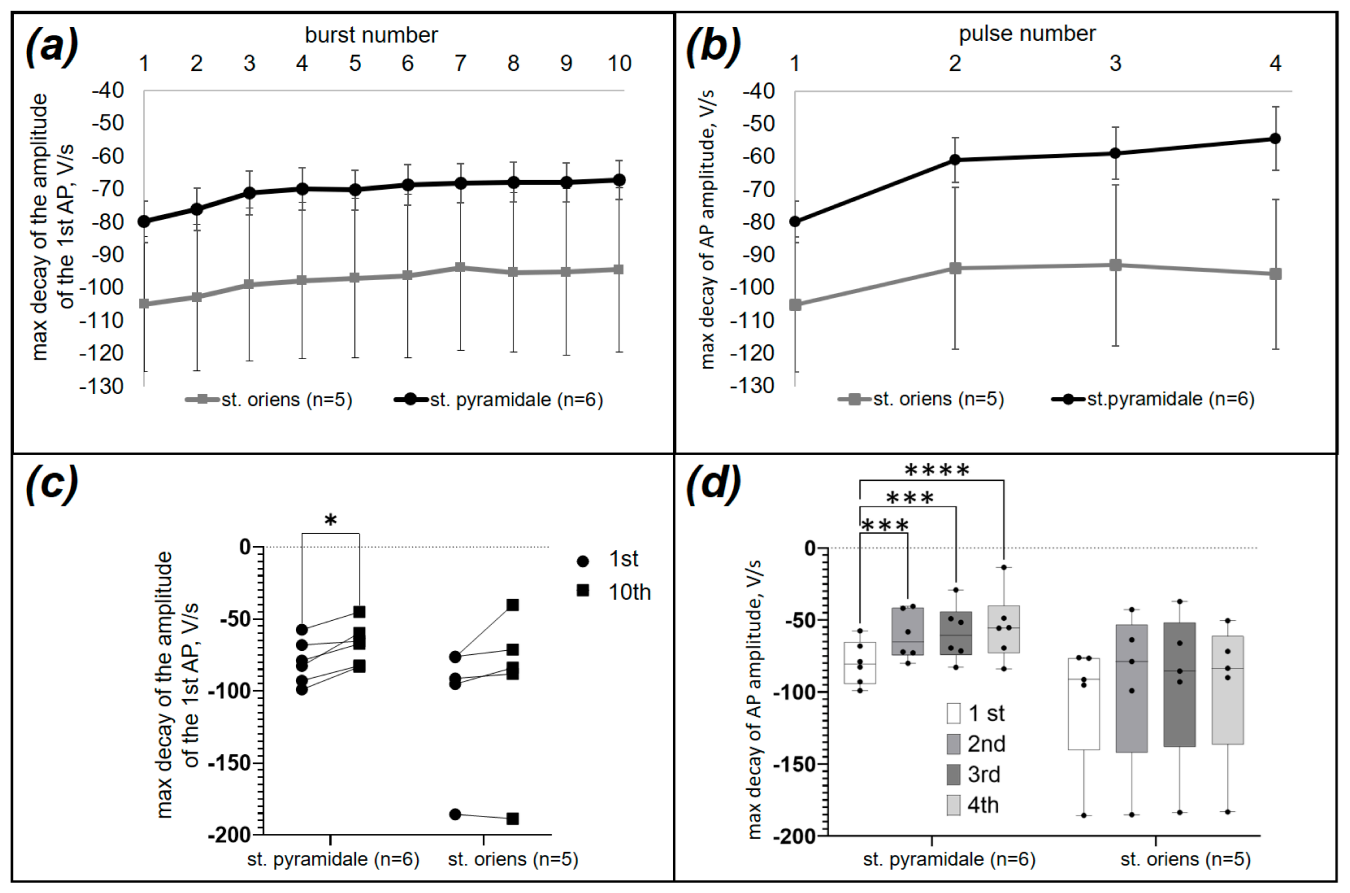
Changes in the maximum decay rate of action potentials in CA1 pyramidal neurons and *stratum oriens* interneurons during the TBS protocol. (**a**) Time course of the maximum decay rate of the 1st action potential across bursts in cells recorded in stratum pyramidale and stratum oriens. (**b**) Changes in the maximum decay rate of action potentials within a burst. Data in (**a**,**b**) are presented as mean ± SEM. (**c**) Individual paired values of the maximum decay rate of the 1st action potential in the 1st and 10th bursts in cells recorded in stratum pyramidale and stratum oriens. Statistical analysis for panel (**c**) was performed using two-way repeated-measures ANOVA followed by uncorrected Fisher’s LSD test; * *p* < 0.05 for the comparison between the 1st and 10th bursts in stratum pyramidale. (**d**) Summary of the maximum decay rate of the 1st–4th action potentials within a burst in cells recorded in stratum pyramidale and stratum oriens. Box plots show the median and interquartile range; whiskers indicate the range, and individual points represent individual slices. Statistical analysis for panel (**d**) was performed using two-way repeated-measures ANOVA followed by the two-stage linear step-up procedure of Benjamini, Krieger and Yekutieli; *** q < 0.001, **** q < 0.0001.

The maximum decay rate of the 1st action potential became less negative during the TBS protocol, but this effect was detected only in cells recorded in *stratum pyramidale* (Figure 8a,c). In cells recorded in *stratum oriens*, the shift of the maximum decay rate of the 1st action potential toward less negative values from the 1st to the 10th burst did not reach statistical significance (Figure 8a,c). Within a burst, the maximum decay rate also became less negative in cells recorded in *stratum pyramidale*, whereas no significant within-burst effect was detected in cells recorded in *stratum oriens* (Figure 8b,d).

**Figure 9:**
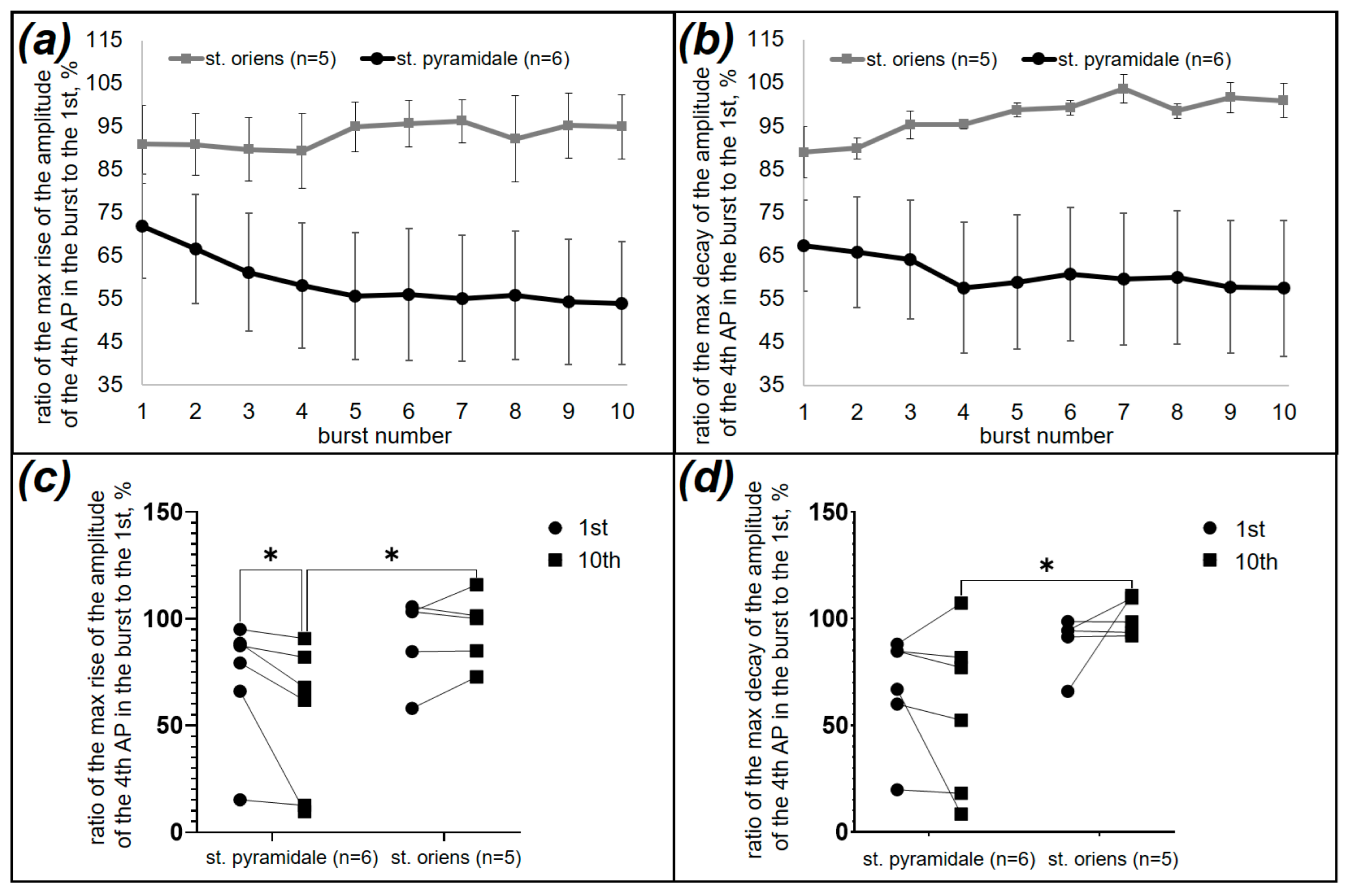
Changes in the ratio of the 4th to the 1st action potential within bursts in CA1 pyramidal neurons and *stratum oriens* interneurons. (**a**,**b**) Time course of the ratio of the maximum rise rate (**a**) and maximum decay rate (**b**) of the 4th action potential to the 1st action potential within each burst during the TBS protocol. Data are shown for cells recorded in stratum pyramidale and stratum oriens and are presented as mean ± SEM. (**c**,**d**) Individual values of the ratio of the maximum rise rate (**c**) and maximum decay rate (**d**) of the 4th action potential to the 1st action potential in the 1st and 10th bursts. Statistical analysis was performed using two-way repeated-measures ANOVA followed by uncorrected Fisher’s LSD test. * *p* < 0.05.

We then analyzed the ratio of the 4th action potential to the 1st action potential within each burst. The ratio of the maximum rise rate of the 4th action potential to the 1st action potential decreased from the 1st to the 10th burst in cells recorded in *stratum pyramidale*, but not in cells recorded in *stratum oriens* (Figure 9a,c). For the maximum decay rate, the ratio of the 4th action potential to the 1st action potential did not show significant depression from the 1st to the 10th burst within either group. However, in the 10th burst, this ratio differed between cells recorded in *stratum pyramidale* and cells recorded in *stratum oriens* (Figure 9b,d).

Thus, whole-cell current-clamp recordings showed that specific parameters of action-potential kinetics changed during the TBS protocol in Ca^2+^-free ACSF. These changes were most consistently detected in CA1 pyramidal neurons. In *stratum oriens* interneurons, within-burst depression and enhancement of within-burst depression from the 1st to the 10th burst were not detected, although a decrease in the first-response maximum rise rate was observed in 3 of 5 cells.

## 3. Discussion

Theta-burst stimulation is widely used to induce long-term potentiation in hippocampal CA1 and to analyze responses that occur during patterned high-frequency activation [5]. In the present study, we used this stimulation protocol under Ca^2+^-free conditions, in which Ca^2+^-triggered neurotransmitter release should be strongly limited [9]. Under these conditions, we recorded a population potential in CA1 that was sensitive to lidocaine, indicating that it depended on the activation of voltage-gated Na^+^ channels. The TBS protocol did not produce detectable post-stimulation changes in the amplitude of this population potential during the 5-min post-TBS recording period. However, several short-term changes were observed during the stimulation itself: depression of the population potential within a burst, depression of the 1st population potential across bursts, and enhancement of within-burst depression during repeated bursts. Whole-cell current-clamp recordings further showed that these population-level effects were accompanied by changes in action-potential kinetics in individual cells, especially in CA1 pyramidal neurons.

### 3.1. Recorded Potential in Ca^2+^-Free ACSF

In Ca^2+^-free ACSF containing 100 μM EGTA, stimulation of the Schaffer collateral pathway did not evoke detectable population excitatory postsynaptic potentials in *stratum radiatum* or synaptically mediated population spikes in *stratum pyramidale*. The potential analyzed in this study was therefore recorded in a different configuration, with the stimulating electrode in CA1 *stratum radiatum* and the recording electrode placed nearby in *stratum pyramidale*. Thus, the stimulus was delivered in the dendritic input layer, whereas the response was recorded from the pyramidal cell body layer.

Extracellular stimulation creates an electrical potential field in the tissue, and the neuronal response depends on how this field polarizes different membrane regions [10]. In *stratum radiatum*, the stimulus could depolarize Schaffer collateral fibers, axon terminals, apical dendritic compartments, and nearby neuronal elements [11]. In CA1 pyramidal neurons, signals arising in the apical dendrites can influence axonal action-potential initiation, and action potentials initiated in or near the axon can propagate back into the dendrites [12,13]. Thus, stimulation in *stratum radiatum* could initiate a local excitation process involving extracellular current spread in the tissue and intracellular propagation along excitable neuronal compartments.

With this electrode configuration, stimulation evoked a brief potential that appeared immediately after the stimulation artifact and decayed within 1–2 ms (Figure 2a). Extracellular fields generated by fast spikes are short, typically less than 2 ms [14]. In our experiments, the potential had a similar time course and was suppressed by 600 μM lidocaine (Figure 2b). Together with the principle that population spikes are formed by extracellular summation of action currents during synchronous action-potential firing [15], these observations support the interpretation of the recorded signal as a brief population response associated with voltage-gated Na^+^ channel activation.

The recorded population potential can therefore be considered an extracellular population-level readout of action-potential-associated activity. It reflects summed action currents generated by synchronously active neuronal elements in the local tissue [14,15]. Such recordings therefore provide a population-level counterpart to current-clamp recordings: fast excitation events are recorded at the level of a local neuronal population.

### 3.2. Ca^2+^-Independent Short-Term Changes During TBS Reflect History-Dependent Excitability

Under Ca^2+^-free conditions, the TBS protocol revealed short-term changes in the population potential during the stimulation itself. These changes were not uniform across the stimulation protocol. The response to a given stimulus depended on the preceding activity: the paired-pulse protocol tested the effect of the immediately preceding stimulus, the interburst series tested the effect of preceding bursts, and the 4th/1st ratio tested whether preceding bursts altered the response structure within a burst. Since the recorded population potential reflects action-potential-associated activity in a local neuronal population, this pattern suggests that the amplitude of the response was shaped by a transient change in the excitability state of the elements that generate synchronous action currents. This interpretation is consistent with the broader view that intrinsic excitability provides a non-synaptic level of regulation of neuronal output [16,17]. Thus, changes in membrane conductances can modify the relation between input and spike output without requiring a change in synaptic strength [16,17]. Our results fit this concept by showing that patterned stimulation can change action-potential-associated output under conditions in which synaptic transmission was not detected.

We hypothesized that the decrease in the amplitude of the population potential within a burst may be related to the refractoriness of voltage-gated Na^+^ channels. To test this assumption, we used a paired-pulse protocol and varied the interstimulus interval from 15 to 1 ms. The amplitude of the second population potential was lower than the amplitude of the 1st potential at all interstimulus intervals, but the pronounced interval-dependent decrease was observed only at intervals less than or equal to 9 ms (Figure 4d). This result defines the shortest temporal component of the effect. After an action potential, excitability is transiently reduced while voltage-gated Na^+^ channels recover from inactivation and K^+^ conductances deactivate [3,7]. Spike propagation can also show short-term history dependence during repetitive activity [3]. Thus, the paired-pulse data support a refractory contribution at the shortest intervals, while the depression observed during 10-ms TBS bursts points to an additional, slower component.

The interburst experiments shift the interpretation from a pairwise refractory effect to a cumulative change in excitability. A statistically significant depression of the 1st potential in the 10th burst compared to the 1st burst was observed only at interburst intervals of 200 and 500 ms, and the response reached a plateau by the 6th burst of stimulation (Figure 5a,c). This temporal profile suggests that the relevant state builds up over several bursts and recovers when the interval between bursts is increased. In CA1 neurons, repeated depolarization can recruit Na^+^ channel inactivation components that recover more slowly than conventional fast inactivation [18,19,20,21]. In particular, intermediate-duration Na^+^ conductance inactivation in CA1 neurons occurs on the scale of hundreds of milliseconds [19], and long-term Na_V_ inactivation in CA1 pyramidal neurons can reduce firing frequency and spike amplitude during trains [22].

The change in the 4th/1st ratio adds another level to this history dependence. The decrease in the amplitudes of the 2nd–4th potentials from the 1st to the 10th burst was more pronounced than the decrease in the amplitude of the 1st potential, so we compared the ratio of the amplitude of the 4th potential to the 1st potential within a burst. This ratio changed only at an interburst interval of 200 ms and gradually recovered when the interval between the 3rd and 4th stimuli in the 10th burst increased from 10 to 100 ms (Figure 5b,d,e). Thus, the preceding bursts changed how strongly the later responses within a burst were depressed. In this operational sense, the enhancement of within-burst depression can be described as a metaplastic change: the earlier activity changed the expression of a subsequent short-term response [17].

Taken together, the TBS protocol separated three Ca^2+^-independent short-term changes in the population potential: depression within a burst, cumulative depression of the 1st potential across bursts, and enhancement of within-burst depression during repeated bursts. These effects were expressed on nested time scales: less than or equal to 9 ms for the strongest paired-pulse interval dependence, 200–500 ms for the cumulative depression across bursts, and 200 ms for the enhancement of within-burst depression. The most consistent interpretation is that TBS transiently shifted the excitability state of the neuronal elements contributing to the population potential. This conclusion naturally led to the next experimental question: whether the same stimulation protocol changes action-potential kinetics in individual neurons.

### 3.3. Whole-Cell Recordings Reveal Predominant Changes in Action-Potential Kinetics in CA1 Pyramidal Neurons During TBS

The population recordings suggested that TBS changed the state of excitable elements contributing to the extracellular response. Whole-cell current-clamp recordings allowed us to test this interpretation at the level of individual neurons. Representative recordings from CA1 pyramidal neurons and *stratum oriens* interneurons illustrate the action-potential responses evoked by the TBS protocol under these conditions (Figure 6). The action-potential waveform reflects the coordinated activity of voltage-gated ion channels, and its rising and repolarizing phases characterize the kinetics of depolarization and repolarization [7,8]. Therefore, we focused on the maximum rise and decay rates of action potentials as cellular readouts of short-term changes in action-potential generation during the same TBS protocol.

In CA1 pyramidal neurons, the maximum rise rate of the 1st action potential decreased from the 1st to the 10th burst (Figure 7a). In *stratum oriens* interneurons, the same tendency was observed only in part of the recorded cells, with a decrease in the first-response maximum rise rate in 3 of 5 cells (Figure 7c). Since the rising phase of an action potential is primarily determined by voltage-gated Na^+^ channel activation, a decrease in maximum rise rate is consistent with reduced availability of Na^+^ conductance during repeated activity [7,22]. In CA1 pyramidal neurons, long-term Na_V_ inactivation was shown to reduce firing frequency and action-potential amplitude during spike trains, and dV/dt was used as a readout related to sodium conductance attenuation [22]. This provides a direct framework for interpreting the depression of maximum rise rate in CA1 pyramidal neurons as a cellular correlate of the short-term depression observed in the population potential.

The comparison between CA1 pyramidal neurons and *stratum oriens* interneurons narrows this interpretation. In hippocampal neurons, Na^+^ channel gating differs between fast-spiking interneurons and CA1 pyramidal neurons: recovery from inactivation is fast and monoexponential in basket cells, whereas in CA1 pyramidal neurons it is slower and contains a slow component [23]. Therefore, the same TBS protocol may affect the depolarizing phase of action potentials differently in CA1 pyramidal neurons and *stratum oriens* interneurons. Within a burst, maximum rise rate decreased across successive action potentials in CA1 pyramidal neurons, whereas this within-burst effect was not detected in *stratum oriens* interneurons (Figure 7b,d).

The maximum decay rate gives complementary information about the repolarizing phase of the action potential. In CA1 pyramidal neurons, action-potential repolarization and frequency-dependent broadening are strongly regulated by voltage-gated K^+^ conductances, including the K_v4.2_-mediated A-type current and K_v2_-mediated delayed rectifier currents [24,25]. In cells recorded in *stratum pyramidale*, the maximum decay rate of the 1st action potential became less negative from the 1st to the 10th burst, indicating slowing of repolarization (Figure 8a,c). This effect is compatible with activity-dependent changes in repolarizing K^+^ conductances or with a broader change in the balance between inward and outward currents during repeated firing [7,24,25]. Within-burst changes in maximum decay rate were also detected in CA1 pyramidal neurons, whereas *stratum oriens* interneurons did not show the same pattern (Figure 8b,d).

The relative stability of action-potential kinetics within TBS bursts in *stratum oriens* interneurons is consistent with known differences between hippocampal principal neurons and interneurons. Fast-spiking interneurons and pyramidal neurons differ in their voltage-gated K^+^ channel composition: fast delayed rectifier K^+^ current predominates in fast-spiking interneurons, whereas A-type K^+^ current is the major component in CA1 pyramidal neurons [26]. K_v3_ conductance is necessary for high-frequency action-potential generation in CA1 oriens-alveus interneurons and is kinetically optimized for repetitive fast firing [27]. These channel differences provide a framework for interpreting why CA1 pyramidal neurons, but not *stratum oriens* interneurons, showed burst-structure-dependent changes in action-potential kinetics during TBS.

The ratio analysis clarified whether repeated bursts changed the within-burst structure of action-potential kinetics. In CA1 pyramidal neurons, the 4th/1st ratio of maximum rise rate changed from the 1st to the 10th burst, indicating enhancement of within-burst depression during repeated stimulation (Figure 9a). This effect was not observed in *stratum oriens* interneurons (Figure 9c). For maximum decay rate, the 4th/1st ratio remained unchanged from the 1st to the 10th burst within each group, although the two groups differed in the 10th burst (Figure 9b,d). Thus, repeated bursts modified the within-burst dynamics of the depolarizing phase mainly in CA1 pyramidal neurons, whereas stratum oriens interneurons did not show enhancement of within-burst depression.

Taken together, the whole-cell recordings support the population-level interpretation of the extracellular data. TBS in Ca^2+^-free ACSF changed action-potential kinetics in individual neurons, with burst-structure-dependent changes expressed predominantly in CA1 pyramidal neurons. *Stratum oriens* interneurons showed a more limited response: a decrease in the first-response maximum rise rate was observed in 3 of 5 cells, whereas within-burst depression and its enhancement from the 1st to the 10th burst were not detected. Thus, the cellular data provide a plausible basis for the depression of the extracellular population potential and show that the Ca^2+^-independent short-term changes observed during TBS are accompanied by changes in action-potential kinetics.

### 3.4. Future Directions for Ca^2+^-Influx-Independent Plasticity

A direct continuation of these findings is to define the membrane mechanisms that generate Ca^2+^-influx-independent changes during the TBS response. The depression of maximum rise rate points to changes in the availability and inactivation state of voltage-gated Na^+^ channels, whereas the slowing of repolarization points to changes in repolarizing K^+^ conductances. This direction can be developed by combining the same TBS protocol with selective pharmacological tools for Na^+^ and K^+^ channels and direct measurements of intracellular Na^+^ dynamics. The same experimental logic can then be applied under conditions with intact synaptic transmission to determine how Ca^2+^-influx-independent membrane plasticity is incorporated into the full response to patterned stimulation.

## 4. Materials and Methods

### 4.1. Ethical Approval

Animal procedures were performed in accordance with the experimental protocol approved by the Bioethical Commission of Moscow State University (protocol code 82.0, 8 June 2017). All efforts were made to reduce the number of animals used and to minimize their stress and discomfort.

### 4.2. Animals

We used adult male F1 C57BL/6×CBA mice, aged 6–10 weeks, obtained from the Department of Functional Genomics, A.N. Belozersky Institute of Physico-Chemical Biology, Lomonosov Moscow State University. Patch clamp experiments were carried out on C57BL/6 mice, aged 4–6 weeks, housed in the animal facility of Pirogov Russian National Research Medical University. Mice were housed under controlled temperature (23 ± 2 °C) and a fixed 12-h light/dark cycle, with *ad libitum* access to food and water.

### 4.3. Extracellular Electrophysiological Recordings

Acute hippocampal slices, 450 μm thick, were prepared manually using a custom-designed vibratome. Following decapitation, the mouse brain was quickly removed and placed in ice-cold, oxygenated (95% O_2_, 5% CO_2_) artificial cerebrospinal fluid (ACSF; in mM: 124.0 NaCl, 4.4 KCl, 1.0 Na_2_HPO_4_, 25.0 NaHCO_3_, 2.0 CaCl_2_, 1.0 MgCl_2_, 10.0 glucose). Then, when all hippocampal slices from the experimental animal were prepared (typically 3–6), they were transferred with a trimmed Pasteur pipette into an incubation chamber. The entire procedure, from animal sacrifice to the transfer of hippocampal slice preparations into the chamber, took no more than 3 min. Slices were allowed to equilibrate in the incubation chamber for at least 1 h before recording evoked electrical activity. In the incubation chamber, the slices were maintained in 200 mL of ACSF at room temperature and continuously oxygenated with carbogen. After incubation, the slices were transferred to the experimental chamber.

We developed a method for recording population neuronal activity in a Ca^2+^-free solution. The solution was prepared based on the composition of artificial cerebrospinal fluid without the addition of Ca^2+^ ions and containing 100 μM EGTA (Ca^2+^-free ACSF). In the experimental chamber, slices were maintained in oxygenated Ca^2+^-free ACSF. A continuous flow of Ca^2+^-free ACSF at a rate of 10–15 mL/min was provided, and the temperature was maintained at 31 °C.

For recording evoked electrical activity, we used a glass capillary filled with a 1.5 M NaCl solution, with a resistance of about 1 mΩ. A silver chloride electrode was placed in the capillary during the experiment. The bipolar stimulating electrode was a double glass capillary filled with Ca^2+^-free ACSF, with silver chloride wires placed inside. To evoke electrical activity, the bipolar stimulating electrode was placed in *stratum radiatum* of CA1, and the recording electrode was placed in *stratum pyramidale* of CA1, in close proximity to the stimulating electrode (Figure 1). The close proximity of the electrodes was necessary due to the fact that in the absence of Ca^2+^ ions, synaptic transmission is impaired; therefore, the propagation of currents in such solution is determined by the electrotonic properties of the cells.

Stimulation control and data acquisition were performed using the WinWCP 5.4.2 program (University of Strathclyde, Glasgow, UK).

Signals were recorded using an amplifier with high-pass (1 Hz) and low-pass (1 kHz) filters; biopotential amplification was 1000×, and the digitization frequency was 1 kHz.

Before the start of the experiment, we positioned the electrodes in a horizontal plane (i.e., relative to the “thickness” of the slice). For this, we selected a stimulus strength of 50 mV, brought the electrodes to the slice, and placed them into the *stratum radiatum* and *stratum pyramidale* layers until the visually maximal response to a fixed stimulus strength was recorded. Stimulation intensity was chosen between 50 and 60% of the maximal response (maximal amplitude of the population potential), determined visually based on testing the maximum and minimum strength before background recording. Stimuli were delivered as pulses with a duration of 0.1 ms every 30 s.

For quantitative data processing, which was performed in Clampfit 10.7 (Axon Instruments, Union City, CA, USA), the population potential amplitude was calculated.

### 4.4. Patch Clamp Recordings

300 µm thick transverse acute hippocampal slices were prepared using a vibratome. The slicing chamber contained an oxygenated ice-cold solution composed of (in mM): K-Gluconate, 140; N-(2-hydroxyethyl)piperazine-N′-ethanesulfonic acid (HEPES), 10; Na-Gluconate, 15; ethylene glycol-bis(2-aminoethyl)-N,N,N′,N′-tetraacetic acid, 0.2; and NaCl, 4 (pH 7.2). Slices were incubated for 30 min at 35 °C before being stored at room temperature in artificial cerebrospinal fluid (ACSF) containing (in mM): NaCl, 125; NaHCO_3_, 25; KCl, 2.5; NaH_2_PO_4_, 1.25; MgCl_2_, 1; CaCl_2_, 2; and d-glucose, 25; bubbled with 95% O_2_ and 5% CO_2_. For recordings, slices were transferred to a recording chamber with continuous perfusion of oxygenated Ca^2+^-free ACSF with 100 µM EGTA at 31 °C.

Patch electrodes were pulled from hard borosilicate capillary glass (flaming/brown micropipette puller, Sutter Instruments, Novato, CA, USA). Electrodes were filled with a solution consisting of (in mM): K-gluconate, 140; HEPES, 10; NaCl, 8; MgATP, 4; NaGTP, 0.3; and phosphocreatine, 10 (pH 7.3 with KOH). Neurons were visually identified using IR-video microscopy. Whole-cell recordings were carried out in current-clamp mode using a EPC 10 USB Patch Clamp Amplifier (Heka Elektronik, Lambrecht, Germany) and the Patchmaster 2x91 software (HEKA Elektronik, Lambrecht, Germany), with a sampling rate of 50 μs.

For intracellular stimulation, the injected current amplitude was chosen as the minimum that evoked an AP on the first depolarization of each burst. Current-clamp intracellular depolarization stimulus duration was 2 ms.

Action-potential kinetics were analyzed on unfiltered recordings using Python 3.9.13 (Python Software Foundation, python.org) with the NumPy 2.0, SciPy 1.13.0, and Pandas 2.2.2 packages. From the first derivative of the membrane potential trace, the maximum positive dV/dt was taken as the maximum rise rate, and the maximum negative dV/dt was taken as the maximum decay rate. Overshoot was taken as the amplitude of the peak of the action potential.

### 4.5. Chemicals

Lidocaine (#L7757) and CaCl_2_ (#SLCD6507) were obtained from Sigma-Aldrich, St. Louis, MO, USA. EGTA (#11290) was purchased from Serva, Heidelberg, Germany; NaCl (#194848) and KCl (#194844) from MP Biomedicals, Santa Ana, CA, USA; MgCl_2_ (#63020) from Honeywell, Charlotte, NC, USA. Na_2_HPO_4_ (#A906159) was obtained from Merck, Darmstadt, Germany. NaHCO_3_ (#6885.2) was obtained from Carl Roth GmbH + Co. KG, Karlsruhe, Germany. D-glucose (#Am-O188-0.5) was obtained from Helicon (Moscow, Russia). Adenosine 5′-triphosphate magnesium salt (#A9187-1G), Phosphocreatine disodium salt hydrate (#P7936-5G), HEPES (#H3375-250G), GTP sodium salt hydrate (#51120-100MG), Potassium gluconate (#P1847-100G), D-gluconic acid sodium salt (Na-Gluconate) (# G9005-500G) were obtained from Sigma-Aldrich, Darmstadt, Germany.

All reagents were of ultra-pure grade.

### 4.6. Statistics

For statistical analysis and graphical representation of data, GraphPad Prism 10.4.0 (GraphPad Software, Boston, MA, USA) and Excel 2016 (Microsoft, Redmond, WA, USA) were used. Data are presented as mean ± SEM unless otherwise stated. Normality was assessed when appropriate. Paired comparisons were analyzed using paired *t*-tests. Comparisons of repeated measures with more than two related conditions were performed using the Friedman test followed by the two-stage linear step-up procedure of Benjamini, Krieger and Yekutieli. Experiments involving two factors were analyzed using two-way repeated-measures ANOVA followed by Tukey’s multiple comparisons test, uncorrected Fisher’s LSD test, or the two-stage linear step-up procedure of Benjamini, Krieger and Yekutieli, as indicated in the corresponding figure legends. Differences were considered statistically significant at *p* < 0.05 or q < 0.05.

## 5. Conclusions

In this study, we showed that patterned stimulation can induce short-term plastic changes in the CA1 region under Ca^2+^-free conditions. The recorded population potential was a brief lidocaine-sensitive response associated with action-potential generation. TBS did not produce detectable post-stimulation changes in this response during the 5-min recording period, but induced depression within a burst, cumulative depression across bursts, and enhancement of within-burst depression during repeated bursts. Whole-cell recordings showed that these population-level changes were accompanied by changes in action-potential kinetics, including depression of the maximum rise rate and slowing of repolarization in CA1 pyramidal neurons.

These findings show that patterned stimulation engages a Ca^2+^-influx-independent level of plasticity that is expressed through changes in action-potential-associated activity. This is important because TBS-induced plasticity is usually interpreted mainly through post-stimulation, Ca^2+^-dependent synaptic mechanisms. Our data indicate that the induction protocol itself should also be considered to be a dynamic process, during which the excitability state of the neuronal elements contributing to the population response is modified. This adds a membrane-level component to the interpretation of patterned stimulation and places intrinsic excitability alongside synaptic efficacy as a relevant substrate of neuronal plasticity. In this sense, the work shows that short-term changes occurring during the induction protocol are not merely a transient background to later synaptic effects, but a measurable form of plasticity in their own right.

## Data Availability

The original contributions presented in this study are included in the article. Further inquiries can be directed to the corresponding author.
